# Psychosocial needs of young breast cancer survivors in Mexico City, Mexico

**DOI:** 10.1371/journal.pone.0197931

**Published:** 2018-05-22

**Authors:** Harper G. Hubbeling, Shoshana M. Rosenberg, Maria Cecilia González-Robledo, Julia G. Cohn, Cynthia Villarreal-Garza, Ann H. Partridge, Felicia M. Knaul

**Affiliations:** 1 Department of Medicine, Harvard Medical School, Boston, Massachusetts, United States of America; 2 Department of Medical Oncology, Dana-Farber Cancer Institute, Boston, Massachusetts, United States of America; 3 Centro de Investigación en Sistemas de Salud, Instituto Nacional de Salud Pública, Cuernavaca, Morelos, Mexico; 4 David Rockefeller Center for Latin American Studies, Harvard University, Boston, Massachusetts, United States of America; 5 Departamento de Investigación y de Tumores Mamarios, Instituto Nacional de Cancerología, Mexico City, Mexico; 6 Centro de Cáncer de Mama, Tecnológico de Monterrey, Monterrey N.L., Mexico; 7 Joven y Fuerte: Programa para la Atención e Investigación de Mujeres Jóvenes con Cáncer de Mama, Mexico City, Mexico; 8 Department of Public Health Sciences, University of Miami Miller School of Medicine, Coral Gables, Florida, United States of America; 9 Institute for Advanced Study of the Americas, University of Miami, Coral Gables, Florida, United States of America; 10 Programa Universalidad y Competitividad en Salud, Fundación Mexicana para la Salud, Mexico City, Mexico; 11 Tómatelo a Pecho, Mexico City, Mexico; Universitetet i Stavanger, NORWAY

## Abstract

**Objective:**

Young breast cancer survivors in Mexico face distinct psychosocial challenges that have not been characterized. This study aims to describe the psychosocial needs of young breast cancer survivors in Mexico at 5 or more years of survivorship, identifying areas of focus for early interventions.

**Methods:**

Breast cancer patients diagnosed at age 40 or prior with 5 or more years since diagnosis were invited to participate in one-on-one 30–60 minute semi-structured audio-recorded interviews at the Instituto Nacional de Cancerología in Mexico City. Transcripts were coded using thematic analysis with NVivo software.

**Results:**

25 women participated. Five major phenomena emerged from analysis: (1) minimization of fertility concerns; (2) persistence of body image disturbance over time; (3) barriers to employment during survivorship; (4) impact on family relationships and social networks; & (5) unmet psychological care and informational needs.

**Conclusions:**

Early interventions with a focus on fertility loss education, access to reconstructive surgery and body image support, guidance during return-to-work, assistance with childcare, integration of psychological care and the fulfillment of informational needs could ameliorate long-term psychological and social distress for young breast cancer survivors in Mexico.

## Introduction

Breast cancer (BC) is the leading cause of cancer mortality and morbidity among women in Mexico and Latin America with 20,444 new cases in Mexico and 152,059 diagnoses in the region in 2012. [1] The burden is increasing, with BC incidence in low- and middle-income countries rising faster than global incidence. [2–3] Compared to other regions, the burden of BC in Mexico is disproportionately borne by younger women; mean age at diagnosis is 52, over a decade younger than in the U.S. [4] Younger women experience lower quality-of-life than older patients in the short- and long-term, with increased illness intrusiveness contributing to psychological distress. [5–6] For young women, diagnosis marks an unexpected shift in life trajectory, often a first experience navigating healthcare, and frequently an isolated one without peers with BC. Compared to older women, young breast cancer survivors (YBCS) are typically more concerned with appearance, more sexually active, and may be in less stable partnered relationships, or seeking a partner. [7] Childbearing plans and professional goals can be disrupted at a critical juncture. [8] Thus, younger women experience BC differently from older women, confronting distinct practical and emotional challenges–challenges which persist years into survivorship.

Several aspects of the health care system in Mexico exacerbate the challenges faced by YBCS. Compounding the inherently more aggressive biologic disease subtypes found in younger women everywhere, delayed diagnosis in Mexico leads to more advanced stage at presentation. [4,9] While 30–40% of BC in the U.S. presents at stage III or IV, this climbs to 50% in Mexico, 58% in Mexican women <40. [10–11] Advanced stage necessitates aggressive treatment–more radical surgery, higher dose radiation, and more aggressive chemotherapy and prolonged hormonal therapy–all of which carry side effects. These side effects–lymphedema, premature menopause, vaginal dryness, cognitive impairment–affect body image, fertility, sexual function, and even employment opportunities, areas critical to the psychosocial wellbeing of young women. In Mexico, options to mitigate some of these side effects are limited: women may not have access to reconstruction after mastectomy, and most facilities do not offer fertility conservation. [12]

YBCS in Latin America remain understudied, limiting abilities to develop appropriate support and treatment strategies. Results from high-income countries are not applicable to low- and middle-income countries, both because of varying treatment patterns and variance in menopause onset making definitions of “young” in high-income countries non-generalizable. Additionally, while the impact of cultural norms on the cancer experience is complex and incompletely understood, cultural context can also limit the applicability of results from studies conducted predominantly in non-Latino white populations. [13,14] For example, fatalism, grossly understood as the belief that individual actions are predetermined by fate, has been identified as a dominant belief among Latinos and is believed to act as a barrier to cancer prevention. [15] On the other hand, equal or superior health outcomes in U.S.-resident Hispanics despite significant socioeconomic and sociocultural adversity–the so called “Hispanic Paradox”–may point to an increased reserve capacity, due to resilience factors including social resources, attitudes such as familism, and religiousness. [16] The experiences of Hispanic/Latino U.S. residents cannot be equated with the experiences of women in Mexico, rather these findings highlight the potential impact of cultural norms on the cancer experience and the need for a dedicated assessment of the psychosocial needs of YBCS in Mexico.

As health system improvements in low- and middle-income countries increase access to treatment through programs such as Mexico’s Seguro Popular, care priorities are being actively redefined and meeting the comprehensive needs of cancer survivors is becoming an increasingly achievable and necessary goal. [17–23] This exploratory study adds to the sparse literature on YBCS beyond high-income countries by describing the psychosocial complications experienced by YBCS in the upper middle-income country of Mexico. The late-stage complications described here identify areas of focus for early interventions that could spare this population from years of BC-related psychosocial distress.

## Materials and methods

### Recruitment

Participants were recruited through the National Cancer Institute (Instituto Nacional de Cancerología, INCan), in Mexico City, Mexico. From July 1^st^ to September 19^th^ 2014, patients enrolled in the study “Exploratory study to associate cognitive state, quality of life, and psychosocial aspects with age in breast cancer survivors” with consecutive medical appointments at INCan were invited in-person by investigators to participate in interviews after their appointments. Eligibility criteria were: female sex, prior diagnosis of stage I-III BC, no known metastases, 5+ years since diagnosis, and age at diagnosis <41. Age cut off was chosen to define a group premenopausal at diagnosis. Comparable studies define “young” as <50 at diagnosis which isolates a premenopausal population in the U.S. (where average age at menopause is 51), but would create a heterogeneous group in Mexico City (where average age at menopause is 46). [24–26] Patients were required to be 5+ years from diagnosis in order to facilitate identification of issues intractable to the survivors’ processes of adjustment and adaptation, as well as to identify later onset psychosocial issues that may arise in the setting of decreased emotional support and narrowing social circles at this point in survivorship. [27–29] Participants were not compensated. The Institutional Review Board of Harvard Medical School (IRB14-1094) and the INCan Ethics and Research Committee approved this study.

### Procedures

Basic demographic and clinical information was obtained from medical records. Participants provided written informed consent and were invited to decline digital audio recording. One-on-one semi-structured interviews were 30–60 minutes long, conducted by one of two female interviewers trained in qualitative interviewing (authors HGH & MGR) in private rooms at INCan. Participants were not acquainted with interviewers prior to interview. The topic guide was developed in collaboration with medical and psycho-oncologists from INCan and informed by exploratory interviews with two young breast cancer survivors (one previously treated at INCan) that were used to generate provisional questions. Open-ended questions were designed to solicit personal illness narratives in keeping with existing literature describing the importance of the patient as a narrative subject and narrative performer. [30–31] The interview guide underwent forward- and back-translation. Data collection was stopped at theoretical saturation. [32]

### Analysis

Audio recordings were transcribed verbatim in Spanish and de-identified by health professionals from Mexico (AABG, CM, JG, RBC, CS, and LOL). Analysis was a multistage process following steps outlined by Strauss and Corbin employing an inductive approach using grounded theory due to the lack of prior literature in this population. [32–33] First, line-by-line review was undertaken by two independent coders using NVivo 10 for Mac (QRS International) to code text fragments in Spanish and identify salient concepts. This was followed by an inductive process of reduction, relation, and abstraction of these concepts using constant and theoretical comparison to establish finely differentiated categories and subcategories organizing concepts. Subsequently, axial coding was used to explore relationships between categories and to relate inductively derived categories to the literature on YBCS. During axial coding, investigators related categories and subcategories at the level of properties and dimensions to create conceptual links and generate hypotheses about the relationships between categories. Coding discrepancies were discussed by coders and resolved by consensus. Ultimately, select categories with associated phenomena felt to be highly impactful for the wellbeing of the study population were chosen for presentation in this manuscript. Select quotes were back-translated to English for inclusion in this manuscript by an independent translator who grew up speaking English and Spanish as co-primary languages (LC).

## Results

Out of a total of 29 women approached, 25 women (86%) were consented to participate in this study. For interviewee demographic and clinical information see Table 1. During inductive coding, five major phenomena emerged: (1) minimization of fertility concerns; (2) persistence of body image disturbance over time; (3) barriers to employment during survivorship; (4) impact on family relationships and social networks; & (5) unmet psychological care and informational needs. Each of these phenomena will be discussed below.

**Table 1:** Demographic and clinical characteristics.

Demographic characteristics		Mean, range *or* fraction (%)
*Age*	*At diagnosis*	36.0, 31–40
	*At interview*	43.4, 37–53
*Marital Status*	*Married at diagnosis*	17/25 (68%)
	*Married at interview*	18/25 (72%)
*Children*	*Children at diagnosis*	19/25 (76%)
	*Children at interview*	19/25 (76%)
*Employment*	*Employed at diagnosis*	18/25 (72%)
	*Employed at interview*	16/25 (64%)
	*Self-employed pre-diagnosis*	5/25 (20%)
	*Self-employed post-diagnosis*	9/25 (36%)
	*Worked throughout treatment*	4/25 (16%)
	*Time off in treatment (years)*	1.5, 0–6
*Economic Status*	*Monthly income (pesos)*	4,288, 1,000–15,000
	*Well off (>10*,*000 pesos/mo)*	3/25 (12%)
	*Stressed (<3*,*000 pesos/mo)*	13/25 (52%)
*Origin*	*Mexico City*	8/25 (32%)
	*Rural Mexico*	17/25 (68%)
*Education*	*Total years of education*	10.9, 4–16
	*University*	8/25 (32%)
	*High school*	6/25 (24%)
	*Primary*	11/25 (44%)
Clinical characteristics		Mean, range *or* fraction (%)
*Stage at diagnosis*	*I-II*	18/25 (72%)
	*III*	7/25 (28%)
*Therapy received*	*Mastectomy with reconstruction*	6/25 (24%)
	*Mastectomy without reconstruction*	15/25 (60%)
	*Breast conserving surgery*	4/25 (16%)
	*Chemotherapy*	24/25 (96%)
	*Radiation*	19/25 (76%)
	*Hormonal Therapy*	15/25 (60%)
*Survivorship (years)*		7.2, 5–14

### Category 1: Fertility

Eleven women reported that they desired more children at diagnosis. Of these 11, six had children prior to diagnosis; five did not. None of these 11 women went on to have children after diagnosis: five did not resume menstruation after treatment, one underwent hysterectomy during treatment, and five described decisions to forgo conception, citing reasons including concerns for a child’s wellbeing (in general and risk of breast cancer) and prioritization of personal health. Study participants rarely independently brought up fertility during interviews; when asked, women did not recount balancing the desire for fertility with survival at any phase of survivorship. When directly questioned about reproductive concerns, patients explained that during diagnosis and treatment their survival took such precedence as to overshadow any thoughts about fertility (see Table 2). Later in survivorship some women described regret and difficulty accepting the reality of infertility. Patients were fearful to broach the topic with providers and not a single woman could recall their physician bringing up fertility in a way that led to a meaningful, informative discussion. Those who did discuss fertility with a physician described conversations limited to warnings to not get pregnant during chemotherapy. Women provided several rationales for this lack of discussion: some felt their fertility concerns could not alter treatment decisions, others emphasized that health took precedence over fertility preservation.

**Table 2:** Quotations representative of Category 1: Fertility.

*Concept*	*Quote (participant #)*
*Concern for health and survival overshadowing fertility concerns*	(11) [Did you want more children?] “As a woman I can say anyone would want more. But, I am no longer interested in that truthfully. What matters to me is my health…” [Were you concerned about fertility?] “No. It never even crossed my mind. The only thing I was concerned with was getting cured.”
	(19) [Did you discuss fertility with your doctor?] “At that moment, the only thing I thought about was my health…”
	(11) “I said cancer, the first thing one thinks of is death, no? What cancer is, I didn’t know. I knew, for me, it was death.”
*Difficulty accepting fertility loss over time*	(10) “My husband said, ‘There is no problem, we have two and that is good. We have a girl, we have a boy, and we are not going to have another one.’ But I said to myself, ‘I want another.’ But it can no longer be. Lately I tell my husband, ‘Hey, we are going to have another baby.’ ‘You’re crazy,’ he says, ‘you’re crazy. You know that you can’t.’”
*Discussing fertility with physicians*	(22) [Are you concerned about fertility?] “To be a mom… I don’t have periods since chemotherapy. I haven’t yet asked the doctors, I’m afraid to ask… My cousin who just got his medical license said it is possible for me to have a baby.”
*Rationalization of infertility*	(20) “Yes, I would have liked to have gotten married, had more children… but, once you have the disease, oh, why bother having children now? Since I do not know if I am going to be able to spend more time with them.”
	(9) [Are you concerned about fertility?] “No, another child? What if it has my same problem? No, no, no.”
	(8) [Did you want more children?] “Before, I used to say ‘I wish I had a son so that he could keep me company.’ Now if I had (had a child) I would have to say, ‘My son is suffering.’”

### Category 2: Body image and intimate partner relationships

Body image narratives were notable for their distinct shapes over time (see Table 3). Some described an initial ‘blow’ to their body image followed by slow adaptation, while others denied body image disturbance at any point. Seven women reported active disturbance at interview including persistent distress since treatment, improvement without complete normalization, and emergence of distress years into survivorship. Active disturbance manifested as a complete loss of interest in appearance or fixation and distress, often pervasive, precluding patients from moving beyond BC. Positive adaptation was often mediated by family members or intimate partners. Specifically, partners provided reassurance that the loss of a breast had no bearing on the couple’s physical or emotional relationship and respect for the woman’s desires pertaining to intimacy. Adaptation narratives often culminated in finding new completeness, or appreciating one’s abilities, framed in reference to those who had lost more, or using a ‘losing x’ versus death dichotomy to minimize loss. Some saw reconstruction as the only way to move on. The five women who did receive reconstruction expressed satisfaction with their physical appearance at interview. Strikingly, one woman reported being told that reconstruction was mandatory due to her young age. Reasons for not undergoing reconstruction after mastectomy included prohibitive expense, risk of infection, poor cosmetic outcomes or reconstruction ‘causing’ recurrence, and uncertainty about how to pursue the procedure.

**Table 3:** Quotations representative of Subcategory 2a: Body image.

*Concept*	*Quote (participant #)*
*Active disturbance at interview*	(17) [Has your self-confidence changed?] “Before the surgery, before all this, I complained about everything. Why am I fat? Why do I have straight hair, not curly hair? Right? Well, when you do not have hair—whatever. And I think we are all the same, women. Except right now, I am not equal to a woman. *crying*”
	(9) [Are you less interested in your physical appearance?] “Yes, I believe that happened. They always tell me, ‘Put make-up on, fix yourself up, do this, do that.’ And I say, ‘What for?’ Yes, I entered that sort of depression.”
	(3) [Are you less interested in your physical appearance?] “Well, no. Because I try to maintain normal self-esteem. I do not like to see myself naked in the mirror. I do not like to see myself without a part of my body. I change without looking at myself. So I look at myself in the mirror only when I have my clothes on or the prosthesis is on.”
*Adaptation over time*	(20) [Have you felt dissatisfied with your body?] “No. Well at first after surgery being without a breast is terrible. But you get used to it, over time. And it really proves that a breast does not prevent you from living life. You see on the metro, many people missing an arm, a leg, blind. I just say, ‘Well, this is something small that doesn’t keep me from walking.’”
	(16) [Was it hard to talk to your kids?] “Yes because I saw myself and I felt bad they had taken my breast. But then, I said, ‘No. Thank God. Because it was taken, I live.’ Then I was assimilating, and now it’s normal for me, that I don’t have my chest.”
	(8) [Could you describe the changes you’ve experienced?] “The change was my breast because the loss made me feel insecure, bad. But I was seeing a psychologist here in the hospital and it lifted my spirits and I got over it.”
	(15) “It happened the moment I saw myself after surgery.. it was at that moment that my mother, a very strong person, made me see: you are not a breast.. ‘I had you without breasts,’ she said, ‘and you are not that, and if you’re alive and if you lose a breast, so what?’ So that’s what got me going, too.”
	(4) “With regards to my children, I was very sorry they saw me without hair. I would put on my wig and my cap. But one day I came out of the bathroom without my hair, completely bald, and my son saw me and I saw him and I said, ‘He’s going to cry.’ But instead, he says, ‘Oh, Lex Luthor!’ That taught me a lot: that there is nothing wrong. My own son taught me, that is the way it is. Since then, I’ve valued many things about my body as well.”
	(9) [Has your self-confidence changed?] “Yes. I feel that now it does not matter so much to be beautiful or dress a specific way. What matters is what you have inside. [More confidence?] More confidence, yes.”
*Reconstruction*	(17) [Describe your mood in the last week] “Good. Nothing. Well, I think for me this is not going to end until.. I think until I get the implant. Because one does feel incomplete, not well. I think that is what prevents you from being in a good mood.”
	(5) [What makes you happiest right now?] “Now I am calm because, despite the depression I have and all that, I feel good. I look good. I had a reconstruction of both breasts so I'm calm. I'm fine with what I have.”
	(8) [Did you need more help?] “I do need someone to help me pay for reconstruction surgery because I do not have the resources truthfully. A doctor raised my spirits because he told me, ‘Don’t be sad, there are reconstructive surgeries available. You’re young and you’re going to get reconstruction.’ But I didn’t know how much it was going to cost.”
	(9) [Did you want reconstruction from the start?] “At first? No. I didn’t think about it, I wanted to be healthy. But now, yes.”
	(3) [Did they ask you if you wanted reconstruction?] “I heard that the most aggressive cancer returned on a reconstructed breast. So I chose not to. I said, ‘I’d better stay this way so it will not happen again.’”
	(5) [Did they ask you if you wanted reconstruction?] “The doctor said it was necessary because of my age . . . I had to reconstruct my breast. There was that doubt in me that said, ‘That’s vanity, I don’t need to do it.’ And the doctor told me, ‘No, you do. You are so young. Why do you want to stay like this? You have to do it and we will.’ So I said ‘Well it’s fine, let’s do it then.’”
	(9) “And then they didn’t reconstruct my breast. [Why not?] They never told me, ‘Hey there’s this, you can do this.’ No. Never. I see that a lot of women did get breast reconstruction, but not me. [Did they ask you if you wanted reconstruction when you had your mastectomy surgery?] No, not at all.”
	(4) “I would’ve liked to have plastic surgery, but my appointments have been canceled. I don’t know if it’s because seven years have passed? Do they give priority to those just getting out of surgery? I don’t know why. There isn’t much communication right now. There is no one to address.”
*Intimate partners and body image*	(3) “To my husband, I would say, ‘Don’t look at me. I don’t want you to see me.’ And he says, ‘Noooo, why? I love you.’ He accepted that I was not naked but wearing a shirt. I didn’t allow him to see me. And he accepted it. That helped me a lot.”
	(10) [Was hair loss difficult?] “No. No. I felt happy without hair and my children and my family, on my husband’s side, would tell me that I was, ‘Uncle Lucas’ and I liked it. My husband said to me, ‘You look pretty,’ and he kissed my scalp.”
	(5) “Seeing my body without breasts, that kind of woke me up to a lot of things, right? Sometimes here is this person with you, but it’s like they really aren’t here with you. Sometimes the person next to you makes you feel like you are alone.”

While many women reported that BC had no effect on their intimate partner relationship, some reported improvement or worsening (see Table 4). One woman’s spouse abandoned her at diagnosis. Many women described increased intimacy and affection as well as appreciation for the lack of social constraint placed upon them by their partners. Withholding intimacy was both a means of rejection and support. Across the spectrum of spousal support, women recognized difficulty talking about cancer. Often this improved with time. Attitudes about dating also evolved over time. Many women single at the time of interview expressed reservations about dating: beginning a new intimate relationship required explaining mastectomy scars, disrupting the façade of ‘normalcy’ women had worked hard to create and protect. Notably, one woman said she could not date because she could not find a partner who would help with illness expenses.

**Table 4:** Quotations representative of Subcategory 2b: Intimate partners.

*Concept*	*Quote (participant #)*
*Adequate support and improved relationships*	(3) [Has your perspective on marriage changed?] “Yes. I value my husband very much because I saw his interest in me. I see his support. When my mom was sick my dad left her. I had that imprinted in my mind, that one has to handle things alone. With my illness, I saw that my husband's support was very different. He never left me.”
	(16) [Has your relationship with your partner changed?] “Yes it has changed. He supports me more in the house and no longer wants me to work so hard. ‘If you want, don’t go to work and stay at home with the children, I will go,’ he says.”
	(3) [Has the support you received made you feel better?] “Yes. Especially my husband. He is very affectionate, very detailed, even the small things. He tells me, ‘Come, let’s go for a walk.’ Or, ‘I’ll buy you an ice cream,’ and then we talk for a long time. It distracts me a lot.”
	(17) [Has the support you received made you feel better?] “When you get the diagnosis you think, ‘This will end our life as a couple.’ Fortunately that was not the case. My husband was here with me.”
*Inadequate support and worsened relationships*	(9) [Do you have a partner?] “When I got cancer, I had my partner, but he left.” [Because of cancer?] Mmhmm.”
	(4) “With my husband, I had many problems. Because I did not have my chest, or out of jealousy, I don’t know. I felt very bad and to date we have managed to fix a lot of things, but not everything.”
	(4) “My husband is a burden to me. It’s more of a weight, but I’m afraid to leave him. I’m scared. He doesn’t help me, but since I lost many things, I lost my parents, my health and everything. The little bit that he does support me, in fact it is very little—for me it is a lot.”
	(5) [Has your partner’s support made you feel better?] “At one point, I needed more company, more closeness and he did not provide that. He was present in another way, bringing me to the hospital, taking care of my medication. But emotionally, I felt he was very far from me.”
	(4) [What are you most concerned about at the moment?] “The safety of my children. If my husband cannot give them that, neither economically or emotionally.. if I get sick, what would happen to them?”
*Intimacy during treatment*	(3) [Has your relationship with your partner changed?] “Since the diagnosis, he has supported me in the sense that he does not sleep with me. He sleeps in another room. At first I said, ‘poor guy.’ But he would tell me, ‘No. Don’t worry. I'm ok. I don’t want to hurt you. I'm afraid that I’m going to move around and I’m going to hurt you.’ In that sense, he has helped me.”
	(5) [Has your relationship with your partner changed?] “He changed a lot because when he was told his wife had cancer, and it was very advanced, that day he took his pillow, his blanket, and left my bed. Then, although he walked beside me for the cancer process and my consults, emotionally he did not. He was as a companion, a person concerned about me, but emotionally, he stepped aside.”
	(18) [Differences from being young?] “My husband has helped me a lot in that respect. He has shown me that it does not matter. We enjoy life sexually with the same intensity, the same lifelong desires.”
*Communication*	(10) “Previously I was not very communicative with my husband or my children. I would go from my work to the house, ‘Are you going to eat?’ ‘Yes’ and that was it. We never sat down to talk, ‘Oh look at this’ etc. Open communication was followed by change in my marriage and my relationship with my children because my husband came closer to me and I to him. He takes care of me and that is the reality.”
	(7) [Do you have a hard time talking about cancer?] “Yes. He didn’t understand my pain very well or my feeling of being mutilated. He started to understand, little by little. He started reading a lot. He’s a man who reads a lot. He investigates.”
	(5) [Do you have a hard time talking about cancer?] “Yes, it has been a lot of work, yes. Now I have to think about how I can bring up the topic. I could not talk about it before at all, no.”
	(21) [Do you have a hard time talking about cancer?] “Right now, they always encourage me, ‘You had cancer, you do not have it anymore. You are not sick; you are as normal as anyone. We don’t want to talk about it anymore.’ There are times when I feel like talking about (cancer) but he does not.”
*Dating*	(20) [Have you felt less or more feminine?] “At first we all feel less feminine because of the treatment, especially losing a breast. You think, ‘Ay! I don’t have a breast, and who is going to look at me?’ Then you think, ‘No. If a person is going to want me, it’s going to be with everything I have.’”
	(4) “I think about how I could possibly have another partner if I don’t have my chest? I don’t know how I could possibly have another partner.”
	(12) [Has your interest in a relationship changed?] “At this stage, I feel like to look for another partner . . . I feel like a partner isn’t going to provide for me because of the disease I have. Because it does come with a lot of expenses.”
	(20) [Is it harder for you to establish a relationship?] “Yes, one is flirtatious, are you not? Haha. The boys always catch one’s eye. But because of this illness you aren’t flirtatious anymore, and then the more you think about it, what’s the point? Especially because I don’t know if I could tell them what I have. So I find it difficult to enter into a relationship. It’s not going to be a normal relationship anymore, is it?”

### Category 3: Employment

Speaking about employment, many women mentioned their children: emphasizing their financial need in terms of dependent children, or describing time occupied by work as taking away parent-child time in the setting of an uncertain future. Patients who took time off work during treatment described their leave as a necessity due to medical appointments, fatigue, or type of work. Patients who continued work throughout treatment unanimously described work as a support–a refuge, useful distraction, or source of normalcy. Of note, three of the four women who were able to continue working through treatment were self-employed. The fourth worked in the formal economy with flexible hours and generous accommodations. Additionally, four women transitioned from the formal to informal workforce after BC. Women employed by family or friends described being able to openly explain their needs and limitations.

Women who had difficulty finding employment after BC described two barriers: lack of accommodation for absences for medical visits and new physical limitations. While many described implicit pressure to not take days off for medical visits, several described overt declarations prohibiting such absences. Several women left housecleaning or factory jobs to avoid heavy lifting after axillary dissections. In particular, patients lacking the education required for non-manual labor jobs described the profound effects of new physical limitations, feeling they had few options left. Many interviewees perceived more general hiring discrimination on the basis of their BC history. Women gave numerous motives for discrimination, namely that employers thought they were not strong enough to work, that they would get sick again, or were there for insurance benefits. Perceived discrimination translated into fear of disclosure: several women chose not to inform prospective employers about their cancer history (see Table 5).

**Table 5:** Quotations representative of Category 3: Employment.

*Concept*	*Quote (participant #)*
*Working throughout as helpful*	(7) [What was your biggest support?] “I took refuge in work, I just worked.”
	(17) [Did you leave your job?] “Well, I have a business, a stationery shop . . . I didn’t leave it, on the contrary, I think that’s what helps me to not think about unpleasant things. I think it was a good distraction. I was trying to have a normal life.”
	(4) [Did you quit your job?] “No. I’m still working. During and after…I dedicate myself to making costumes. I had to make a lot of chicken costumes, for kindergarten, I had three days to deliver these costumes and I still had to sew in all the little faces. I brought them and I sewed in eyes and noses during chemotherapy and a nurse told me, ‘Oh nice, to distract yourself.’ I said, ‘If you only knew it was an emergency,’ haha. That helped me. It helped me be active and not be ‘sick.’”
*Young children*	(14) [Are you worried about being fired?] “Sometimes, yes. I worry because my daughters are still in school. I wouldn’t say they are little girls, but they do still need me.”
	(20) [Did you have to quit your job?] “…It has taken me a long time to get myself back to work. I now dedicate a lot more to my child because.. the truth is, with this, no one knows when, right? So I spend more time with my little girl and work somewhere where I don’t get absorbed for a long time. A job where you don’t have to be around all day.”
*Medical appointments*	(10) [Was it difficult to find a job?] “I like to be honest when I apply for a job. I say, ‘I have cancer and I have to go to therapies and consults.’ They tell me, ‘We will not grant you permission.’ ‘Okay. Perfect. Thank you very much and see you later.’”
	(8) [Was it difficult to find a job?] “Yes it was difficult because, in reality, since I have to go to my appointments, not everyone says, ‘Oh yes, go ahead and take a day off.’”
*Physical limitations*	(8) “My life changed completely. Because my arm doesn’t work 100% as it did before because of the nodes removed. My doctor said it couldn’t carry half a kilo anymore because I run the risk of it getting inflamed if I force it. So since I don’t have an education, I have to do factory work or things like that, and then, yes, it’s more complicated, but, well, there I go.”
	(9) [Things to make life easier?] “Employment. Work is not easy to come by. And with my hand, well . . . If there were programs that could help us financially and teach us to do things to get ahead . . . that will help us find employment.”
*Perceived hiring discrimination*	(10) [Have you returned to work?] “No. No one wants to hire a cancer patient. We are no good to them. Yes, discrimination. We are no use; we no longer have the strength to do the job. Yes, they see it like that. So, they no longer hire us.”
	(9) [Has it been difficult to find a job?] “With my problem, nobody will give me a job. [Why?] Because I have a history of cancer. They don’t say it out loud, but it’s true. It is very difficult for a cancer patient to get a job. It is as if one is no longer of any use, or if one becomes ill, they assume one will just abandon the job. But no, they no longer give us work. So here I am with my mom, washing dishes.”
	(16) [Has it been hard to find a job?] “..If you have this disease, they no longer want to hire you because they think you only came for the insurance and benefits.”
	(23) “‘We will call you,’ (potential employers) say, then the medical record arrives and they never speak to me again. It fills me with impotence and desperation… I am a woman who likes to work.”
*Fear of disclosure*	(20) [Do you feel there is hiring discrimination?] “…I don’t think I would tell anyone that I had.. I had experienced this. [Due to fear?] Exactly. Maybe I won’t say it.”
	(20) [How have co-workers reacted?] “Oh, I honestly haven’t talked to them about my illness. I feel it’s something private.”

### Category 4: Family and social networks

Concern for children and family members, often supplanting concern for self, was a common narrative (see Table 6). During early illness, physical separation of families resulted in significant strain. Separation resulted from women from rural areas moving to Mexico City for treatment or from husbands moving away to send back funds. Patients who commuted to and from treatment, particularly single mothers or women without spousal support, were stressed by having to leave young children (or elderly parents) waiting outside during treatments. Other sources of stress related to children were fear that daughters would get BC and uncertainty regarding what, when, and how to tell children about BC. Many women found ways to conceal their illness experience from children. Yet often women were surprised by how well their children reacted. Older children stepped up to care for siblings and a traditional arrangement with the mother responsible for all household tasks in several cases yielded to a more equal division of labor between all family members, persisting even after the mother recovered from treatment.

**Table 6:** Quotations representative of Subcategory 4a: Family.

*Concept*	*Quote (participant #)*
*Family separation*	(15) “This has had a negative impact on my children. I was always with them. Then the moment they diagnosed me and I came to the hospital, I left them with their dad. Then my children were in the streets, doing things they didn’t do before. And my husband also started playing cards. He didn’t gamble before. It is as if it is all my fault.”
	(17) [Things to make life easier?] “. . .To be able to not to worry about my family. Going through chemo and knowing that my mom is out there, she’s worried. I think you care more about those outside and not the ones inside because we know we are being taken care of. To think she had to wait so many hours. I was most worried about that, ahhh but she HAD to come.”
	(4) “I didn’t have anyone to leave my children with. I brought them here and they were in the car, they ate breakfast in the sun and I was here in chemotherapy. I think childcare is very important because I was worried that my kids were out there.”
*Worrying about family more than self*	(6) “Well, my family became very sad. They suffered more than I did. When you have control, you do not suffer. But seeing other people suffer, that is what worried me.”
	(4) [Requests for your medical team?] “. . .The husband is also affected by this. One usually thinks, ‘Oh poor her,’ but the husband also suffers. The husband should also receive counseling along with the wife.”
	(3) [Things to make life easier?] “…My children were aware of the disease. It has been very difficult for them. My son, the middle one, I saw him living in fear. When I slept, he would put a mirror under my nose to see if I was breathing. I saw the anguish in him. If he had not been like this, I wouldn’t have.. my anguish was more for him than for me.”
	(14) “My mom also had cancer and it runs in her side of the family. I wish there were places where our daughters could go to get diagnosed early and prevent this type of disease.”
*How much to tell children*	(19) [Was it difficult to tell your children?] “Yes. They had to know. Sooner or later, they had to know.”
	(20) [Was it difficult to tell your children?] “Yes, it was difficult. In fact, he doesn’t know what I have. He only knows I’m going to the doctor. But the disease itself, I didn’t have to tell him. It wasn’t necessary for him to know.”
	(21) [How did your kids react?] “As children they didn’t know what it was, if it was bad or good, and therefore if it was something normal and nothing else. They listened to me and understood. But now they are older and understand more [Is it harder to talk to them now?] Yes, it is difficult because you don’t know how to start the conversation with them.”
	(17) “I think my children didn’t realize the effects of chemotherapy. Because what I tried to do was to get up early, get them ready for school, then when they left for school, I went to bed. So when they got back, I was a little better.”
*Positive effects on children*	(14) [Was it hard to talk about cancer with your kids?] “I never thought my sons were going to react in that way: to grow, value life. They matured faster than usual.”
	(18) “It was difficult to cope with the time I wasn’t with them. But this test gave us a union, a maturity. I have wonderful children because they see life differently. Maybe because they experienced this trial, they don’t hang out in the streets. They are children who are very close to their family with many principles; maybe (cancer) made my children that way.”
*Redistribution of household chores*	(6) [How about household chores?] “My husband helped me more around the house and now he has taken ownership of those chores because I couldn’t do some things while I was being treated. He started doing them, and kept doing them.”
	(14) [How about household chores?] “Before, they left everything to me. I had to go here and there. But not now, right now, everyone does something and if somebody cannot do something, the rest take over.”

Beyond family, many found that illness permanently narrowed their social circles to ‘true’ friends only (see Table 7). Two woman described becoming very socially isolated post treatment. And while one interviewee denied the existence of stigma, most experienced stigma from their community in some form: several women encountered people who thought cancer was contagious. Women presented opposing attitudes about partaking in the BC patient community: some shied away from other patients in an effort to view their experience as distinct while others found strength communing with other patients. Interestingly, many felt strongly compelled to assume the role of “patient advocate” as part of a new identity in recovery. Many knew peers who had passed away, and these deaths often informed personal survivorship identity, either as cause to reject or embrace the label of ‘survivor’.

**Table 7:** Quotations representative of Subcategory 4b: Social networks.

*Concept*	*Quote (participant #)*
*Real friends emerging*, *others disappearing*	(7) [Types of reactions?] “There are people who are with me to this day, celebrate every win with me, year after year, in this battle with cancer. Those who are Catholic pray and those who are yogis emit good vibes, haha! I learned after my illness to surround myself with people who support me. Those who didn’t, well, I lost their friendship, period. I have no grudges against them.”
	(6) [Types of reactions?] “Well, not all my friends gave me support. Some became distant for fear of how I might deteriorate. They told me after the fact, they said, ‘I was afraid to see you. I thought I was going to find you, I don’t even know how.’”
*Isolation*	(5) [Do you meet up with your friends like before?] “Well, no. Not much. Not as much because I have another vision of life. I don’t know. I became isolated. I liked to have friends; I really liked to talk. But now I like being alone and walking alone, seeing the forest, park—alone. I like thinking alone. After I recovered, that’s how I became, very isolated from everyone.”
	(23) “It’s really difficult for me to approach people. The words don’t come out of my mouth, it’s like there is a knot in my throat. Everything passes through my mind.. but it is like I swallowed a spider web down in there. Yes, I endured the treatment.. I don’t know why after all that, it’s now that I’m becoming this fearful, I feel very bad.”
	(23) “..I don’t know what is happening to me. Even I don’t understand it. I used to be very sociable, I went out with friends, with my family. Now I stay in my house and I don’t leave for anything, I don’t go out to eat–nothing. I don’t understand.”
*Stigma*	(21) “There are many people who can’t understand what cancer is. They think if you get near them, they’ll catch it.”
	(9) [Have you faced stigma?] “Yes, I do feel that I have, yes. If you go to a party, if you don’t drink, you are ‘this.’ If you don’t smoke, you are ‘that.’ If you want to go to bed early, you are a drag, the one who doesn’t want to be here. But only you know how far you can go.”
	(14) [Have you faced stigma?] “I believe you yourself make people reject you . . .people’s rejection is provoked by you. There is no such kind of rejection.”
*Patient community*	(21) “I saw others sick with BC and thought, ‘I’m going to get like that.’ But, no. My doctor explained -each person is different.”
	(18) “At first I didn’t want to talk about it. As a matter of fact, when I was here during the course of my treatment, I didn’t like to talk to anyone because they started saying, ‘That's what happened to my family.’ I didn’t want to imagine that was what was happening to me. I knew I was a different person with a different cancer and I preferred at best, saying, ‘Good afternoon,’ but not talking about cancer. Because I felt like I was going to create mountains in my mind.”
	(14) “The young ladies of the ‘Challenges’ group taught us many good things. Truthfully, they did help me a lot spiritually, with the strength, will, and discipline that one needs to get out of that hole into which so many of us fall.”
*Identity as a patient advocate*	(14) [How do you see your future?] “Well, with my daughters, relatives, sisters, I advise them to constantly check themselves. I try to encourage the people I know not to be afraid of operations or treatments or those difficult tests that are required, like biopsies or mammography. [Do you consider yourself recovered?] Yes, I see myself as recovered and with the desire to move on and be a person that can be approached with questions so that they can see in me that one can survive.”
	(18) “At first, people were morbid, ‘Ay she has cancer,’ and so on. But then I got over it. That doesn’t affect me. On the contrary, I talk about it so people start getting tested early.”
	(17) [Has your life perspective changed?] “Yes, it has changed me and I feel that something else still needs to be done. Maybe having been given this precise life experience, I think I’m still not in a position to give encouragement and motivation to others, because I lack these myself on most days, however . . . this is not going to end until it ends.”
*Other patients dying*	(9) [Do you consider yourself a survivor?] “After I came to the hospital I met five girlfriends . . . Of those five, only two are left . . . The other three, who were younger, have died. How could I not consider myself a survivor? Of course I do, thank God.”
	(21) [Do you consider yourself a survivor?] “I’m still afraid because the patients who have had the disease longer, they tell us that in five, six years at best, it comes back somewhere else.”
	(7) [Things that would’ve made life easier?] “That after I got sick two great friends would not have gotten sick and they would not have died of this. They were diagnosed with cancer after I was and I motivated them and they did not *crying* It is difficult because you lose hope. You see yourself as someone who could survive, but two of your friends could not. You ask, ‘Why? What am I doing here now?’”

### Category 5: Psychological care and informational needs

Many women spoke of an unmet need for psychological care (see Table 8). Two recounted suicidal thoughts: one passive, one with active ideation and plan. Some women described intense anxiety during the transition to survivorship care as they received less frequent visits and scans, worrying that their disease would come back, undetected. Additionally with yearly appointments, women felt out of touch with support groups and other programs at the hospital. In contrast, other women described repulsion to the hospital, vomiting as they entered, or feeling unable to walk through the door. The two patients who saw psychiatrists described immense benefit, even from very few sessions. Barriers to receiving psychological care included cost, time, and uncertainty about how to find a provider.

**Table 8:** Quotations representative of Subcategory 5a: Psychological care.

*Concept*	*Quote (participant #)*
*Psychological distress*	(11) “It was very unpleasant news when I got the diagnosis. When I left, I felt bad at that moment. I felt very badly. Believe me when I say that I didn’t care if I got hit by a car in that moment. I don’t care, I told myself, that way I would die in one go.”
	(8) “I cried a lot, a lot, a lot. I would say, ‘Doctor, it’s just that I feel very bad.’ I thought about suicide. I said I’ll just grab a gun and kill myself and I’ll be done. Because it was so much pain and I didn’t know how the process would go with the medicines, everything that was going to happen. I didn’t know anything about that. I said, ‘Cancer, well I'm going to die either way. I won’t be cured of this disease. Why not just kill myself now and get it all over with, no?”
	(14) “I was bathing and I put soap in my hair. When I went to remove the soap, all the hair came off on my face and I couldn’t breathe. I got really nervous, yes, a phobia of not being able to breathe and from seeing my hair fall out. So that caused me a lot of havoc.. I had to call my husband to help me out of the bathroom because I was drowning.. it was a trauma.”
	(17) [Describe the changes you’ve observed since diagnosis?] “I still cannot overcome this. *crying* Three years have passed since I finished treatment and—no. I still think this will never be over.”
	(17) “I still feel sorry for myself and I can’t get over this. [Is there something that could help you?] Well, no. I don’t think so. [Does time help?] Well, no. No. When you get home after surgery, you say, ‘It has to happen, doesn’t it? It has to happen. Days will pass and I'm going to be better.’ And right now, for example, five years have passed, waiting for this to change. I tear up easily now, I cry all the time.”
*Anxiety at the transition to survivorship*	(6) [Ways to improve your care?] “. . . During checkups, you always have this nagging feeling, right? Right now, I come once a year and I feel that is too long. I’d rather come every month and be safe. A lot can happen in a year.”
	(21) [Ways to improve your care?] “Let me have an MRI every year to see what cancer now affects my body. Because with a mammogram of only the breast and nothing else, I won’t know if I have cancer somewhere else. Also, give us a copy of every exam done here so we have that information ourselves.”
	(21) “I come from very far away and then they do nothing else but review the tests we were sent to do and a physical exam and that’s it. It would make us feel more confident if they did a little more.”
	(10) “I felt a little anxious, anguished. Because I’m thinking, ‘They’re not treating me like they used to treat me. The disease is going to come back and I won’t know.’ Why? Because I'm not going to the doctor every eight days. I'm not going to get checked on. Yes, it gave me anguish and I felt so worried. Because they gave me appointments every month, then two months, then three months, four, five and now six. So now I go every six months and I still feel distressed by the same thing that I was seen more often and not anymore. The anguish is for everything. Even if we say, ‘We are healed, we are cured of the disease,’ and, ‘I am no longer afraid of that,’ there is also that possibility, that anguish, biting at us that the disease can come back and can get you, that it isn’t going to end.”
	(9) “There should be more help for those of us who, since we are only seen once each year, often we don’t know what is available, what new programs there are for us here at the hospital.”
*Distress entering the hospital*	(8) “It was so bad that when I entered the hospital, it was already starting, *retching impression.* I hadn’t begun my treatment and I was *retching impression.* It was psychological.”
	(11) [Things that would’ve made life easier?] “It would’ve been easier if I didn’t have to be in this place. The truth is, this is an unpleasant place. This place breaks my soul, because I already went through it. I can’t see patients like this because it breaks my soul to see them because I already lived it. I feel horrible for their families.”
	(14) “One comes and goes from here with fear because simply arriving, the smell of the chemotherapies . . . it’s almost like cats. I would get to the door, but I didn’t want to go in any further. The door was open but, ‘not any further, not any further.’”
*Explicit requests for psychological care*	(4) [Ask for two things from your medical team] “After ten years, after eight, ask the patient how she feels, how she is doing. Above all they should make it like a law that they send in the psychologist with the other consults: chemo, radiation, and a psychologist. Make it the default. I think that is very important because I think my disease really didn’t affect me. It really was a small tumor and they got it out and that’s it! But, the psychological part… The physical effects, well, yes, the chemo gives you a disgusting feeling and you feel bad and all that. But the fear, your family, your children, the morale… that hurts terribly. Yes, you should see it as a special department that exists so women are cared for psychologically and have a better quality of life. We are being cured, physically, but what becomes of our minds? How are we psychologically?”
	(4) “I’ve been thinking a lot about going to the psychologist. I don’t have the opportunity right now because of work and everything. But I have felt very sad. I feel like going away, living elsewhere. I’ve really been feeling that I want to leave.”
	(4) [Things that would’ve made life easier?] “You are naive when you first get cancer. If I had known then what I know now, I wouldn’t have worried so much. If I had been in a group, like the ‘Challenges’ group, or if there had been something like this interview where they asked me how it was going, or if I had gone to a psycho-oncologist it would have made it easier.”
*Psychological care as helpful*	(4) “The psychologist said a few words that made things very clear. I went a few times. I asked him, ‘After cancer, what will my life be like?’ I didn’t know. I was still being treated. I asked if I was going to die and he said, ‘Not today.’ ‘More people die from accidents than cancer.’ And I said, ‘How can I avoid this? It’s killing me, the idea that I have cancer.’ And he tells me, ‘One has to learn to live with this. Learn that your whole life you’ll be coming to the hospital. All your life you will see sick people. It’s a way of life.’ So that helped me a lot to understand what was happening to me. I went to five sessions and I came to understand: why am I distressed if I have to come here the rest of my life anyway, right? You have to accept it.”
	(8) “I cried and I told my doctor, ‘I feel very bad, I feel very bad. I feel that I will not be able to go on’ …And he sent me to the psychologist and the psychologist told me, ‘No. This is a disease like any other and you have to make it. But if you are sad and you cry and you don’t eat and you are suffering, the medicine will not work. You have depression and 50% of this is hope and 50% is the medication. So right now cry all you want to cry and then you’re not going to cry anymore.’ And so I cried and cried and cried. But then I said, ‘No.’ One day, just like that, I said, ‘That was enough. I have to get better.’”

Notably, the one patient with active suicidal ideation described her motivation in terms of information deficit: desperation rooted in a lack of understanding of what medicine could do for her. In general, patients reported that unmet informational needs contributed to their anxiety. They desired more information on topics including navigating the hospital’s administrative process, reconstruction, nutrition, lifestyle, where to seek non-oncological care, and side effects such as lymphedema and hormonal changes (see Table 9). While the majority of informational needs pertained to early survivorship, patients also identified needs later on (e.g. where to seek gynecologic care fifteen years out). Patients frequently described filling information gaps with rumors heard in the waiting room. The representative quotations in Table 9 provide a sense of the breadth of topics women desired more information about and the extent to which women felt having more information would ameliorate their distress.

**Table 9:** Quotations representative of Subcategory 5b: Informational needs.

*Concept*	*Quote (participant #)*
*Non-oncology providers*	(6) “Well, I can say that when you talk to others who suffered from the same (disease), it generates doubts. Because they tell you that you need to come to this hospital right away for any illness. Actually I was never told that by my doctor, so I get treated elsewhere when it’s not related to my cancer. But maybe you should be more careful, for example, with something like pap smears, maybe you should go to someone who is a specialist?” (18) [Ways to improve your care?] “Maybe I’m a bit reserved, not so questioning, but a lot of things I learned from the outside. How I should eat, what I shouldn’t do. I have a good doctor but she isn’t here in oncology, she’s a general practitioner. She supports me a lot better, gives me a little more detailed information as to what I need to do, what care one needs to take when they remove a part of your body. That kind of thing.”
*Informational needs during treatment*	(6) [Things that would’ve made life easier?] “One arrives here very fearful and you don’t know how to do things properly.. At first I would run all over the place and I was very worried. I didn’t know the hospital. At first we needed a little more help with all the logistics, the administrative things, sometimes you have no idea how to do them and you are afraid.”
	(3) “My nails fell off. I was very fond of them. It didn’t hurt, but they started to secrete fluids and then came off my feet and hands. It traumatized me. That was what affected me the most, more than when my hair fell off. I think it would help if we were kept more informed of the changes that were going to happen to our bodies. I didn’t know that your nails could fall off. I expected my hair to fall off, eyebrows, but I didn’t expect it to escalate to my nails. When I had an opportunity, I asked the doctor and she explained it. That is when I was reassured that it was normal.”
	(9) [Ways to improve your care?] “When one comes out of surgery, they don’t tell us that we are at risk. I used a hammer and my arm became very inflamed. Yes, more information.”
*General desire for information*	(17) [Ways to improve your care?] “Be a little more explicit. Let’s talk a little more. More information. We do what they tell us and we know we are with the specialists, but there are things that seem silly, doubts when we don’t know what is coming next or why something is happening.”

Overall, the categories presented above–fertility, body image & intimate partner relationships, employment, family & social networks, and psychological care & informational needs–organize important concepts expressed by this group of YBCS in Mexico relating to their psychosocial wellness, which we will now attempt to situate within the YBCS literature, generating suggestions for the survivorship care of this growing population.

## Discussion

### Fertility

The most prominent finding pertaining to fertility was, in fact, the category’s lack of prominence. [34–36] We attribute this to several factors. First, regional discrepancies in average maternal age result in fewer patients in Mexico with zero children at diagnosis and thus more moderate infertility-related distress than what is reported from high-income countries. [37] Second, in the setting of low- and middle-income countries where survival may be less presumed, a survival-first mentality overshadows fertility concerns. [38] A third factor is patient-physician dynamics. Patient accounts here are consistent with suboptimal rates of fertility discussion by physicians as documented in a prior study in Mexico as well as in the U.S., and globally. [12,39–40] The belief expressed here that fertility concerns could not influence treatment choices could be due to inadequate information provision by physicians, or poor recall of patient-physician conversations. Of note, a significant minority (8–29%) of BC patients choose less aggressive treatment in favor of fertility preservation, demonstrating knowledge of–and engagement in–treatment choices that was not described in this cohort. [36,41] Finally, minimization of fertility concerns can aid in coping. [34,42] Yet, minimization should not be equated with true lack of impact on the survivorship experience. In fact, minimization of fertility concerns coexisted here with infertility-related depressive symptoms and distress persisting years into survivorship. [43]

### Body image and intimate partner relationships

The persistent, severe, body image-related distress seen in this cohort lasting years into survivorship is unfortunately not a novel finding. [44–45] However, it remains concerning, particularly in light of the strong correlation between poor body image and depression in Mexican women. [46] The qualitative design of this study allowed insight into the wide range of trajectories of body image perception over time, and provides some clues about what can steer that course. [47] The phenomenon of family positively mediating body image seems overrepresented in these experiences compared to survivors in other countries, which may be due to the distinct cultural role of the wife/mother in Mexico. [48–49] The lower body image satisfaction and elevated distress seen here among women who desired but could not receive reconstruction is consistent with findings of YBCS literature from high-income countries. [50] While our findings highlight limited access to reconstruction due to expense and process uncertainty, they also illustrate the complexity of decision-making surrounding reconstruction, with women weighing numerous concerns (e.g. infection risk, poor cosmetic outcome, sense of wholeness).

With regard to intimate partners, while the interdependent distress, anxiety, poor communication and even outright emotional and physical abandonment presented here cannot be ignored, stories of intimate partner relationships were largely remarkable for their resiliency and growth. [51–54] In contrast, we did not find any examples of dating as a positive experience capable of restoring self and bodily esteem, which has been described in the literature. [7,55] While this absence could be due to small sample size, it could also be reflective of cultural factors modulating the profoundly vulnerable experience of physically and verbally revealing a BC history to a new partner.

### Employment

Participants’ attitudes regarding return-to-work experiences largely matched findings in the international literature. Echoing respondents in European and U.S. studies, most participants in this study characterized working as a coping strategy, providing a sense of purpose and normalcy. [56] In contrast, the women who reported valuing work less as they came to view working as time not spent with their children exhibited a reprioritization of work-life balance that has also been documented in BC scholarship. [8] However, it is important to note that in this cohort, the lasting financial impact of BC made de-prioritization of work impossible for many women.

In contrast to the concerns about unaccommodating workplaces and perceived discrimination seen here, most studies in the U.S. and Europe report accommodating supervisors and supportive work environments. [57] However, analyses in high-income countries focusing on weak workplace support do show that employer sensitivity, employer-employee communication, and perceived willingness of the employer to accommodate needs can affect whether or not women return to work after a BC-related absence. [58–59] In this study, concerns about absences for medical appointments often precluded women from getting jobs in the first place. Mexico’s large informal economy (22.6% of GDP in 2016) seems to have provided additional employment opportunities and flexibility for some women. [60] However, the informal sector, largely a manual labor sphere, does not offer a solution for all YBCS. The disproportionate difficulty of returning to work for lower income, less educated survivors, for whom new physical limitations precluding manual labor can be devastating, were particularly well-illustrated in our study. [61]

### Family and social networks

Social relationships dominated patient narratives both in terms of the frequency of mention and length of discussion and women frequently equated social wellbeing with overall wellbeing. [62] In general, young children were focal to the experiences of these YBCS be it as sources of anguish or strength. Women employed all three of Adam’s coping strategies grappling with raising young children during illness, most unique to this population being the ‘balancing’ act of needing to travel far away for treatment and not wanting to be apart from young children at such a time. [34] Patients’ concern for family above self became a means of ‘normalizing’: holding on to a provider role instead of transitioning to a self-focused ‘sick role’. [63] And while women normalized family interactions in the emotional domain, they described permitting, and benefiting from, a more pragmatic ‘change’ process: long-term redistribution of household chores.

In contrast, beyond the household, we found predominantly negative perspectives on how broader social circles interacted with women’s BC experience: narrowing of social circles, social isolation, and fear of disclosing BC status. [64–65] Stories of social isolation reported here are valuable in their depiction of the complete aloneness some patients find themselves in after treatment, something not well understood through the quantitative literature. It is interesting that the desire to become a mentor figure to other patients was very prevalent in this group. This adds to the literature’s emphasis on patients’ desires for mentorship during treatment, and could represent a ‘changing’ process; as women adapt to survivorship, becoming a mentor to other patients gives positive, new, meaning to broader social interactions. [66]

### Psychological care and informational needs

While depressive symptoms have been shown, in general, to taper with time from diagnosis, younger women are at higher risk of long-term depression, as exemplified by the pervasive anguish present at 5+ years survivorship. [6,67] As seen here and in the literature, the transition to survivorship can be a period of elevated psychological distress. [68] Studies in the U.S. show that Latinos underutilize mental health services in comparison to other ethnic groups, often attributing this to the stigmatization of mental health (e.g. saying ‘nervios’ to refer to mental health problems in order to avoid ‘socially damaging’ clinical diagnoses like depression or anxiety). [69] While evidence from Mexico is lacking and the generalizability of findings in Latinos is debatable, it is notable that several patients here explicitly argued for more integration of psychological care, suggesting that, in some cases, patients would readily embrace, not shy away from, such services if they were made more available.

Causes of the information deficits observed here include inadequate communication with healthcare providers, as well as a deficiency of written and electronic information on issues faced by young patients, such as fertility preservation, genetic counseling, and treatment side effects. [70]

### Limitations

While our participation rate was high, non-response bias could still have led to missed perspectives. Furthermore, the survival bias inherent in interviewing only women without recurrence at 5+ years survivorship means that our results do not describe the experiences of women who may recur early in their survivorship. Additionally, and importantly, while speaking to women at 5+ years after diagnosis gave critical insight into women’s understanding and narration of their survivorship experience at a potentially reflective point and allowed identification of needs persistent in late survivorship, asking these women about experiences they had years prior introduces recall bias and undoubtedly overlooks acute psychosocial needs that YBCS confront in the immediate post-treatment period. Finally, in order to solicit longitudinal narratives of survivorship and adaptation processes, our interview guide directly asked about “changes,” possibly at the cost of minimizing narratives of consistency of experience over time.

## Conclusions

In light of the recent implementation of universal health coverage in Mexico with Seguro Popular, and the subsequent expansion of access to early diagnosis and treatment of BC, we see an opportunity for the development of a national plan for BC control, one including comprehensive supportive care integrating early palliative and psychosocial interventions to prevent complications later in survivorship. This study’s rich qualitative view into the challenges of Mexico’s YBCS over time points to several important components of such an intervention.

First, we suggest emphasizing early discussion of fertility loss, ideally pre-treatment. Patients must be made aware of the possible impact of treatment on fertility regardless of limited options available for fertility preservation on site. Providers discussing fertility with YBCS should keep in mind the often transient survival-first mentality of early phase survivors as well as patients’ fear of bringing up fertility-related concerns. Ultimately, steps should be taken to ensure that physicians are equipped to adequately discuss fertility and that patients and survivors feel informed after these conversations. Second, available financial assistance should be used to make reconstructive surgery more broadly accessible and emphasis placed on thoroughly addressing patient concerns about surgical treatment and honoring informed patient choice. All patients, particularly those denied reconstruction, should be invited to participate in interventions targeting body image early in treatment. Third, occupational therapy programs and educational materials on employment after BC should be provided, especially to women previously employed in manual labor with a financial imperative to return to work. Further work is needed to assess and address possible employment discrimination against cancer survivors in Mexico. Fourth, day care programs at cancer centers and/or family transportation and lodging should be provided (as financially possible) to minimize distress related to physical separation from children and elder relatives during treatment.

Finally, mental health care, currently not part of oncologic care in Mexico, must be integrated into, and emphasized, in survivorship care. Formal assessment and therapy with psycho-oncologists should be routine. Additionally, we strongly believe in the benefit of survivor-advocate programs, designed to address both emotional and informational needs. Such programs can improve the social, psychological and even financial wellbeing of survivors both in the roles of mentees and mentors. Women may be more willing to ask questions, and more able to retain information in the setting of a peer group than in their doctor’s office. And by creating a social space where the YBCS experience is normalized, such groups can provide a forum for healthy processing of many of the concepts presented above, guard against the complete social isolation reported here, and enable YBCS not only to tell their BC story, but to shape their story and use it: “so that they can see in me that one can survive”.
